# Physical Interaction between Cyclin-Dependent Kinase 5 (CDK5) and Clock Factors Affects the Circadian Rhythmicity in Peripheral Oscillators

**DOI:** 10.3390/clockssleep4010017

**Published:** 2022-03-09

**Authors:** Jürgen A. Ripperger, Rohit Chavan, Urs Albrecht, Andrea Brenna

**Affiliations:** 1 Department of Biology, Faculty of Science and Medicine, University of Fribourg, 1700 Fribourg, Switzerland; juergenalexandereduard.ripperger@unifr.ch (J.A.R.); rohitchavan27@gmail.com (R.C.); urs.albrecht@unifr.ch (U.A.); 2 Laboratory of Cardiovascular and Aging Research, Department of Endocrinology, Metabolism, Cardiovascular System, Faculty of Science and Medicine, University of Fribourg, 1700 Fribourg, Switzerland

**Keywords:** CDK5 circadian, mouse embryonic fibroblasts, phosphorylation, period length

## Abstract

Circadian rhythms are self-sustained oscillators with a period of 24 h that is based on the output of transcriptional and post-translational feedback loops. Phosphorylation is considered one of the most important post-translational modifications affecting rhythmicity from cyanobacteria to mammals. For example, the lack of cyclin-dependent kinase 5 (CDK5) shortened the period length of the circadian oscillator in the Suprachiasmatic Nuclei (SCN) of mice via the destabilization of the PERIOD 2 (PER2) protein. Here, we show that CDK5 kinase activity and its interaction with clock components, including PER2 and CLOCK, varied over time in mouse embryonic fibroblast cells. Furthermore, the deletion of *Cdk5* from cells resulted in a prolonged period and shifted the transcription of clock-controlled genes by about 2 to 4 h with a simple delay of chromatin binding of ARNTL (BMAL1) CLOCK. Taken together, our data indicate that CDK5 is critically involved in regulating the circadian clock in vitro at the molecular level.

## 1. Introduction

Circadian rhythms consist of interconnected transcriptional/translational and post-translational feedback loops that dictate the rhythmicity of physiological responses within a 24 h period [1,2]. These rhythms can be synchronized by stimuli called zeitgebers (ZT). For instance, in mammals, light can reset the rhythmicity in the suprachiasmatic nuclei (SCN), a bipartite brain region belonging to the hypothalamus, placed above the optic chiasm. Thus, the SCN maintains the internal rhythm in synchrony with the external dark-light cycles [3]. In addition, the SCN coordinates the synchronization of the independent circadian oscillators situated in peripheral tissues (i.e., liver, kidney, heart) through neuronal and hormonal signaling pathways, regulating a wide range of physiological responses [4]. Rhythmic transcription is driven by the core factors of the positive loop, the heterodimer ARNTL (BMAL1): CLOCK. This heterodimer promotes the expression of many clock-controlled genes (CCGs), such as *Period* (*Per 1*, *2*, *3*) and *Cryptochrome* (*Cry 1*, *2*), by binding to the E-box element (CACGTG) within their promoter [5]. The PER2: CRY1 homo- or heterodimer is the core element of the negative loop, ensuring gene expression rhythmicity [6,7]. After protein translation, PER: CRY1 homo/heterodimers translocate into the nucleus and inhibit ARNTL (BMAL1): CLOCK–mediated transcription through direct protein-protein interaction [8]. Thereby, the cellular positive and negative feedback loop influences the oscillation of about 10–20% of all genes expressed [9,10]. Additionally, the fine-tuned period of the circadian oscillators is ensured by many additional levels of regulation, such as epigenetic and post-translational modifications (PMTs) [11,12]. Within the PMTs, phosphorylation is the most critical factor affecting rhythmicity [13]. In some organisms, such as cyanobacteria, the circadian rhythmicity can be driven only by phosphorylation without the need for transcription or translation [14].

In mammals, many kinases regulate circadian rhythms [2]. Cyclin-dependent kinase 5 (CDK5) is a serine/threonine kinase belonging to the CDC2/CDK1 family activated by specific cofactors such as p35 and p39 [15,16] and cyclin I [17]. CDK5 plays many roles in the brain, including neurogenesis, neuronal migration, axon guidance, and aberrant activity leading to neurodegenerative diseases [18]. Recently, it has been shown that CDK5 regulates the main circadian clock. CDK5 can phosphorylate both CLOCK and PER2 [19,20]. This kinase influences PER2 protein stability and interaction with CRY1 in the SCN [20].

Lack of CDK5 shortens the circadian period length in mice [20]. Therefore, this kinase has been proposed as one of the central regulators of circadian rhythmicity in mice [2]. In addition, many recent observations led to the discovery of the involvement of CDK5 pathways outside the brain. CDK5 regulates vesicular transport, apoptosis, cell adhesion, and migration [21]. Dysfunction of CDK5 in peripheral tissues is associated with many forms of cancer and is also connected to the aberration of the circadian clock [22,23].

Here, we analyzed the involvement of CDK5 in the regulation of peripheral clocks, using immortalized mouse embryonic fibroblasts as a model system. Our results showed that deletion of this enzyme led to a prolonged period length measured by real-time luciferase assays. In addition, the kinase activity of CDK5 was circadian, with a peak during the subjective night. Deleting the *Cdk5* gene, which resulted in the depletion of CDK5 protein, was associated with an aberrant shuttling of clock factors into the nucleus. Thus, ARNTL (BMAL1) and CLOCK recruitment to an E-box of *Per1* was shifted, altering gene expression. Altogether, our results verify that CDK5 is essential for the correct timing of the clock machinery.

## 2. Results

### 2.1. Lack of CDK5 Lengthens the Period Length of a Peripheral Clock

**Figure 1:**
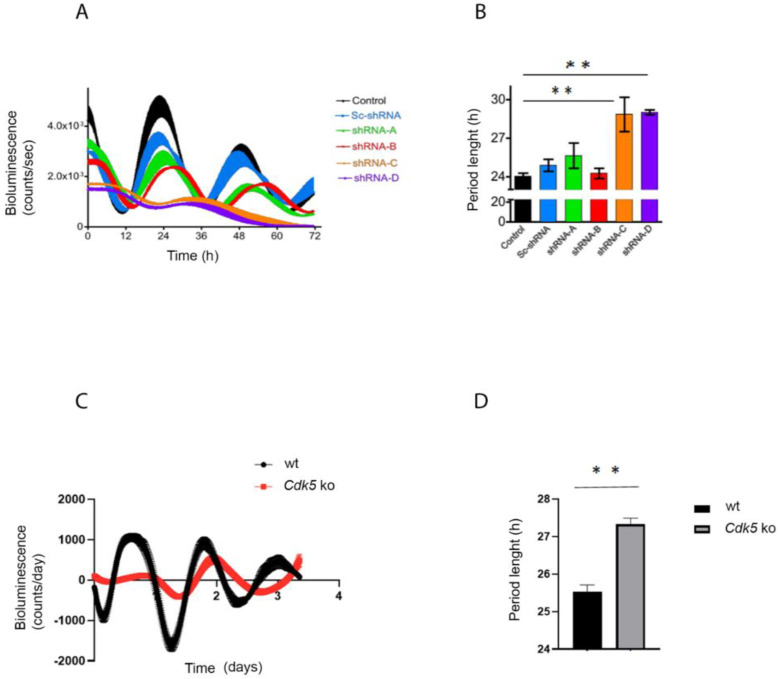
CDK5 affects the circadian clock. (**A**) NIH 3T3 cells were transfected with different shRNA against *Cdk5*. Forty-eight hours after the silencing, cells were transfected with an *Arntl*^luc^ reporter construct and synchronized with 100 nM dexamethasone. The bioluminescence was recorded over several days. (**B**) The period length was quantified, and statistical analysis was performed. The results showed that silencing of *Cdk5* could prolong the period length. Quantification of three independent experiments (*n* = 3, mean ± SD) was performed. One-way ANOVA with Bonferroni’s post-test, *: *p* < 0.001. (**C**) Wild-type and *Cdk5* ko cells were transfected with an *Arntl*^luc^ reporter construct and synchronized with 10 µM forskolin. The bioluminescence was recorded over several days. (**D**) The period length was quantified, and statistical analysis was performed. The results showed that the absence of CDK5 in *Cdk5* ko cells could prolong the period length. Quantification of three independent experiments (*n* = 3, mean ± SD) was performed. One-way ANOVA with Bonferroni’s post-test, *: *p* < 0.001.

We transfected NIH 3T3 with an *Arntl*^luc^ (*Bmal*1^luc^) reporter construct and treated cells either with DMSO (vehicle) or roscovitine (general CDK inhibitor; [24]) at different concentrations for 12 h. This treatment was followed by synchronizing the cells with 100 nM dexamethasone, a general in vitro circadian synchronizer, followed by recording the real-time luciferase bioluminescence over 3 days (Figure S1A). NIH 3T3 cell lines treated with DMSO showed a regular period length, which settled around 23 h, while an increasing amount of the drug corresponded to a prolonged period length (Figure S1B). To investigate the direct involvement of CDK5, we subsequently modulated the *Cdk5* gene expression transfecting the *Arntl*^luc^ (*Bmal1*^luc^) reporter construct in NIH 3T3 cell lines with either a control or four different shRNA expressing constructs against *Cdk5* mRNA described before [20] (Figure S1A). Two of the tested knock-down constructs, shRNA-C and shRNA-D, which corresponded to the more robust silencing of *Cdk5*, could lengthen the period of the *Arntl* promoter-driven luciferase reporter (Figure 1A,B). The construct shRNA-D was used to knock-down *Cdk5* expression in vivo [20]. Additionally, the effect of knock-down of *Cdk5* on the period length was similar to the dose-dependent action of roscovitine (Figure S1A). 

Finally, deletion of *Cdk5* (CRISPR/Cas9 *Cdk5* ko cells, [20]) induced the same phenotype as the knock-down or roscovitine treatment, resulting in a more extended period (Figure 1C,D). Hence, we must conclude that it was not simply the knock-down construct that caused the discrepancy between the shorter period obtained in vivo and the longer obtained in vitro. Together, our results prove that our in vitro model system is reliable in showing that a lack of CDK5 is associated with a prolonged circadian rhythm. 

### 2.2. The Kinase Activity of CDK5 Is Circadian in Mouse Embryonic Fibroblasts

**Figure 2:**
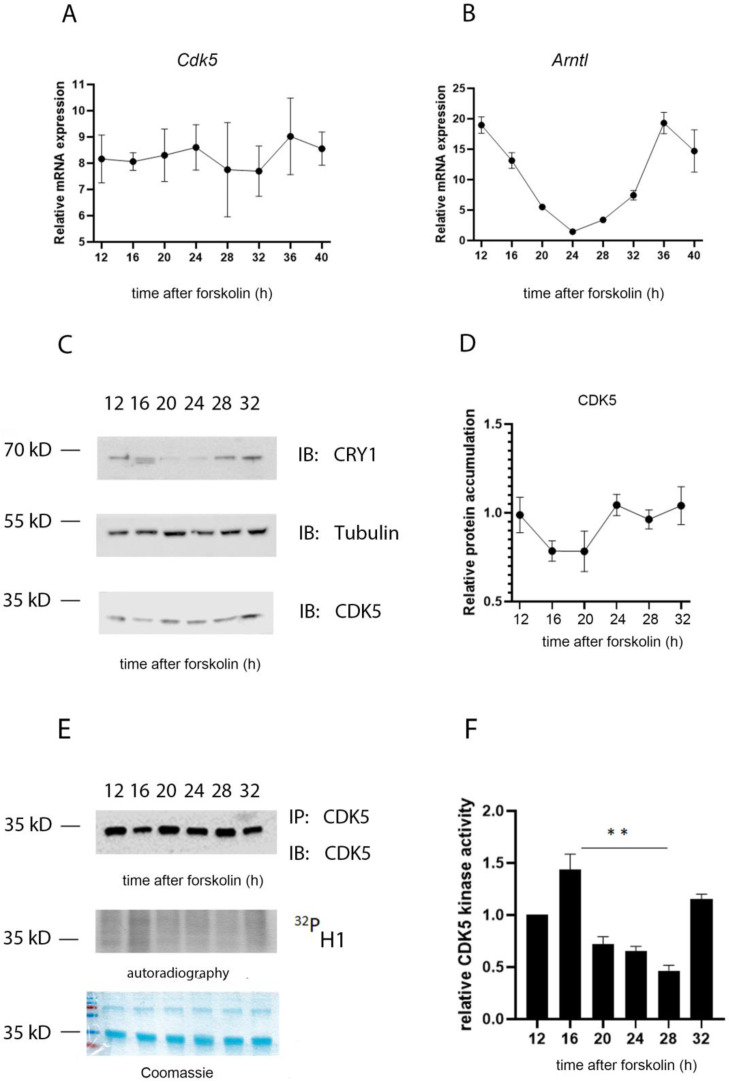
CDK5 kinase activity is circadian. The temporal profile of mRNA induction for *Cdk5* (**A**) and *Arntl* (**B**) was studied in forskolin-synchronized wild-type cells collected at different time points. While *Cdk5* gene expression was constant over time, *Arntl* was oscillating as expected. Quantification of three independent experiments (*n* = 3, mean ± SD) was performed. (**C**) Western blot analysis was performed on forskolin-synchronized wild-type cells collected at different time points. To validate the circadian cellular synchronization, samples were detected by WB analysis with an antibody against CRY1. CDK5 detection showed an accumulation over 24 h with mild oscillation. Tubulin was used as a loading control. kD: kiloDalton. (**D**) Quantification of three independent experiments (CDK5/Tub, *n* = 3, mean ± SD) was performed. (**E**) CDK5 was immunoprecipitated at different time points after forskolin synchronization and, subsequently, an in vitro kinase assay was performed using histone H1 as substrate in the presence of radiolabeled γ^32^P^-^ATP. (**F**) The radioactive signal was normalized on the immunoblotted signal for CDK5 after the IP. Quantification of three independent experiments (*n* = 3, mean ± SD) was performed. One-way ANOVA with Bonferroni’s post-test, *: *p* < 0.001.

Due to the impact of CDK5 on circadian rhythmicity, our first question was whether *Cdk5* gene expression and/or protein accumulation were also rhythmic. We used NIH 3T3 mouse embryonic fibroblast cells as a model system to address this question. We synchronized the cells with 10 µM forskolin, a powerful zeitgeber to induce circadian rhythmicity [25], and collected samples at different time points after induction. We performed RT-qPCR on reverse-transcribed RNA collected every 4 h starting from 12 to 32 h after forskolin treatment (FST). Our results showed that the expression of *Cdk5* was relatively stable throughout the experiment (Figure 2A). *Arntl* gene expression, which showed a clear oscillation, was used as a control for the efficiency of the forskolin entrainment of the cells (Figure 2B). We subsequently tested whether CDK5 protein accumulation was oscillating. To this intent, we performed a Western blot (Wb) analysis of protein extracts obtained at the same time points as in Figure 2A. First, we validated the forskolin-mediated circadian entrainment of cells by immunoblotting against CRY1. CRY1 protein accumulation showed a robust circadian oscillation, confirming that the cells were adequately entrained (Figure 2C). By contrast, CDK5 protein accumulation showed only a shallow rhythm (Figure 2C,D).

Previously, it was found that the CDK5 kinase activity in the SCN is diurnal, with an off and on state under light and dark (12:12) conditions, respectively [20]. To understand whether this activity in NIH 3T3 cells was circadian, CDK5 was immunoprecipitated at each time point from synchronized cellular extracts, followed by an in vitro kinase assay using histone H1 as substrate in the presence of radiolabeled [γ-^32^P]-ATP. The autoradiography signal showed that CDK5 kinase activity is circadian with an “on” state at 12, 16, and 32 h after FST (Figure 2E, F). These time points would correspond to the subjective night of an animal, supporting the observation obtained from SCN samples [20]. The specificity of the reaction was verified by repeating the assay by immunoprecipitating the kinase from *Cdk5* knock-out (ko) cell lines (negative control) or wild-type (wt) cells pretreated with 35 µM of the CDKs inhibitor roscovitine [26], followed by an in vitro kinase assay. The phosphorylation of H1 occurred only in the sample of the wild-type cells not treated with the inhibitor (Figure S2). These observations strengthen the hypothesis that CDK5 kinase activity is regulated circadian but not protein accumulation.

### 2.3. CDK5 Is Part of the Clock Machinery

**Figure 3:**
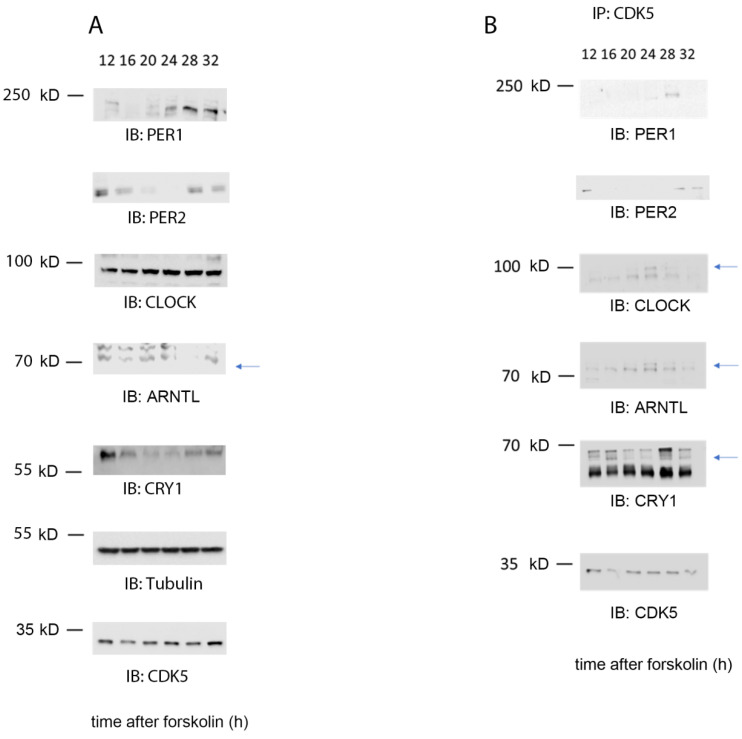
CDK5 is part of the clock machinery. (**A**) Western blot analysis was performed on total extract proteins collected from NIH 3T3 cells at different time points. (**B**) Immunoprecipitation was performed around the clock on wild-type cells synchronized with 10 µ forskolin using CDK5 antibody as a bait. As a result, CDK5 co-immunoprecipitated with ARNTL (BMAL1), CLOCK, CRY1, PER1, and PER2 at specific time points. Blue arrows: specific signal.

CDK5 can influence circadian rhythmicity in animals and cells and physically interact with CLOCK and PER2 [19,20]. Therefore, we questioned whether this kinase could be part of the clock machinery. However, many examples of kinases being part of the complex in central and peripheral clocks were described before, such as Casein kinases (CKs), AKT, or GSK3β kinases [2,27]. Therefore, we synchronized cells with 10 µM forskolin and collected samples at different time points, starting 12 to 32 h after FST. WBanalysis confirmed the proper synchronization of cells by probing the samples with antibodies against PER1, PER2, CLOCK, ARNTL (BMAL1), CRY1, CDK5, and tubulin as a loading control (Figure 3A). Subsequently, we immunoprecipitated CDK5 every 4 h starting from 12 to 32 h after FST, followed by immunodetection of CLOCK, ARNTL (BMAL1), PER1/2, and CRY1 (Figure 3B). Our results showed that CDK5 could interact with PER1 at 24–28 h after FST and PER2 at 12, 28, and 32 h. Similarly, CRY1 interacted with CDK5 at 12, 16, 28, and 32 h after FST. Interestingly, ARNTL and CLOCK interacted with CDK5 during the subjective day, with an identical profile at 20 h and a more robust interaction at 24 h after FST. This observation supports the idea that CLOCK and ARNTL work as heterodimers [28]. Altogether, our results show that CDK5 interacts with multiple parts of the clock machinery.

### 2.4. CDK5 Influences the Nuclear Localization of Clock Factors

**Figure 4:**
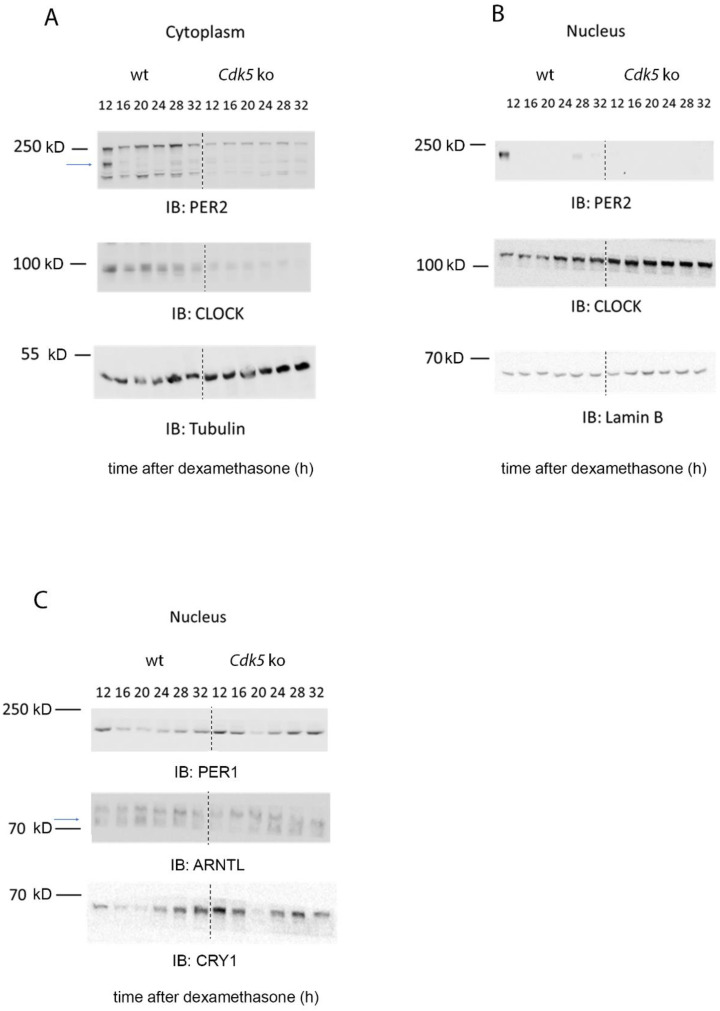
CDK5 influences the nuclear localization of clock factors. (**A**) Western blot analysis of cytoplasmic extracts obtained from wild-type and *Cdk5* ko cells, showing how the kinase affected the circadian accumulation of CLOCK and PER2. (**B**) Western blot analysis of nuclear extracts obtained from wild-type and *Cdk5* ko cells, showing how the kinase affected the circadian accumulation of CLOCK and PER2. (**C**) Western blot analysis of nuclear extract obtained from wild-type and *Cdk5* ko cells, showing how the kinase affected the circadian accumulation of ARTL (BMAL1), CRY1, and PER1. Tubulin was used as a cytoplasmic marker, while Lamin B was used as a nuclear marker.

So far, only CLOCK and PER2 are direct targets of CDK5 phosphorylation [19,20]. Therefore, we investigated whether PER2 and CLOCK accumulation and distribution in the NIH 3T3 cells could be affected without the kinase. Cells were synchronized with 100 nM dexamethasone and collected at different times. This sample collection was followed by nuclear/cytoplasmic fractionation to observe whether the cellular distribution of CLOCK and PER2 was affected by CDK5. In control cell lines, we could detect PER2 at 12, 16, 28, and 32 h after dexamethasone in the cytoplasmic fraction, while detection occurred at 12, 28, and 32 h after dexamethasone in the nuclear fraction (Figure 4A,B). On the other hand, PER2 was observed in a reduced amount only 12 h after dexamethasone in *Cdk5* ko cells in the cytoplasm, while the signal was practically absent in the nuclear extract (Figure 4A,B). These results support previous observations showing that CDK5 is essential for PER2 protein stability and nuclear localization [20]. Subsequently, we analyzed CLOCK cellular distribution. The cytoplasmic protein accumulation showed a flat profile, while the nuclear accumulation was rhythmic, peaking between 24–32 h after the stimulus in wild-type cells (Figure 4A,B). By contrast, cytoplasmic accumulation of CLOCK was dampened in *Cdk5* ko cells lines. CLOCK was strongly accumulated in the nuclei, without any rhythmicity, and the protein level was more abundant than in the nucleus of wild-type cells (Figure 4A,B). Altogether, these observations indicate that CDK5 had an opposite effect on CLOCK compared to PER2 in murine embryonic fibroblasts. 

Both CLOCK and PER2 are primary members of the molecular clock machinery [27]. Additionally, we showed that CDK5 is part of this complex (Figure 3). Thus, we sought to investigate how the lack of CDK5 could affect the nuclear accumulation of other proteins that are parts of the machinery, such as ARNTL (BMAL1), CRY1, and PER1. As a result, our immunoblotting showed that ARNTL accumulated in the wild-type nuclei at 20 h after dexamethasone, while the protein accumulation was shifted by about 4 h in the *Cdk5* ko cells (Figure 4C, blue arrow). Furthermore, immunoblotting for PER1 and CRY1 showed that in wild-type cell lines, the nuclear accumulation of these factors was higher at the subjective night. By contrast, the nuclear accumulation of these proteins in the *Cdk5* ko cell lines followed the same profile of wild-type cells, but the protein detection was much more robust and visible also during the subjective day (Figure 4C). Taken together, our results showed that CDK5 influences the temporal and spatial cellular localization of the clock machinery in mouse embryonic fibroblasts. 

### 2.5. Delay of RNA Rhythmicity due to the Lack of CDK5 Is Mediated by a Shift of ARNTL and CLOCK Binding to Chromatin

**Figure 5:**
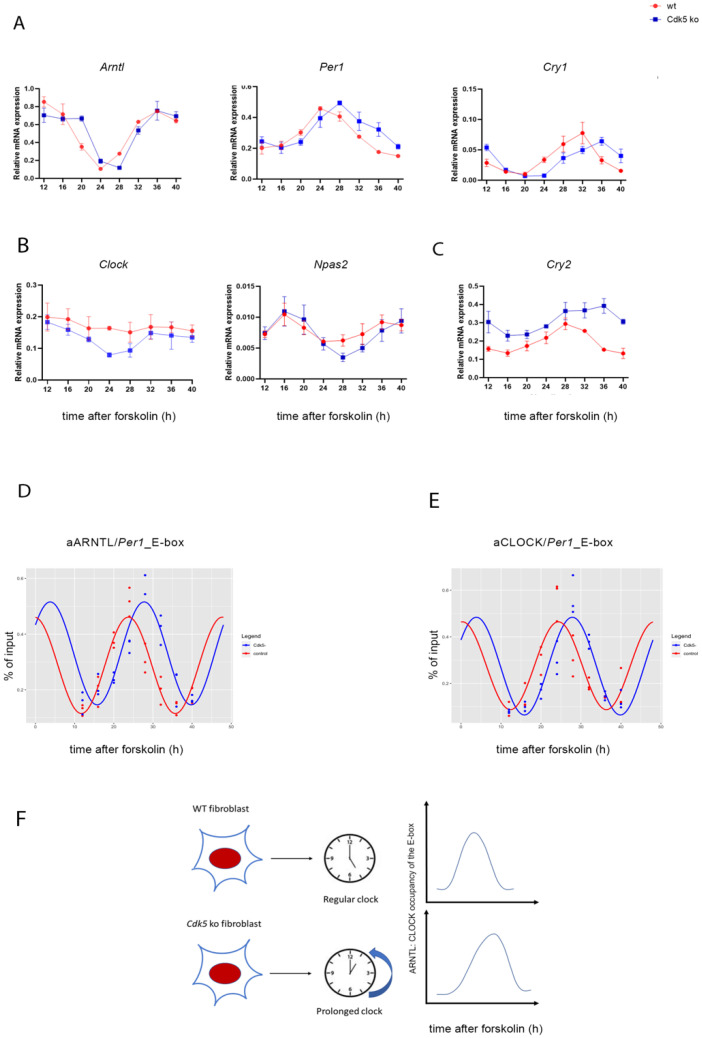
CDK5 influences the expression of clock-controlled genes and affects CLOCK: ARNTL (BMAL1) promoter occupancy. (**A**–**C**) The temporal profile of mRNA accumulation of many clock-controlled genes was analyzed in synchronized wild-type and *Cdk5* ko cells. Quantification of three independent experiments (*n* = 3, mean ± SD) was performed. One-way ANOVA with Bonferroni’s post-test, *: *p* < 0.001. Chromatin immunoprecipitation was performed using ARNTL (**D**) or CLOCK (**E**) antibody as bait. RT-qPCR was performed using specific probes that amplified the E-box region of *Per1*. Quantification of three independent experiments (*n* = 3, mean ± SD) was performed. Rhythmicity was analyzed using CircaCompare [31]. For ARNTL [∆(*Cdk5* ko-wt]: ∆mesor = −0.043 (AU, n.s.); ∆amplitude = −0.011 (AU, n.s.); ∆phase = −4.04 (h, *p* < 0.001). For CLOCK [∆(*Cdk5* ko-wt]: ∆mesor = −0.002 (AU, n.s.); ∆amplitude = −0.018 (AU, n.s.); ∆phase = −3.49 (h, *p* < 0.001). (**F**) Summary showing that lack of CDK5 can prolong the period length, delay ARNTL and CLOCK recruitment to E-boxes, and affect circadian gene expression.

Our results showed that CDK5 is part of the clock machinery, and the lack of this enzyme was responsible for the spatial and temporal redistribution of the circadian clock complex. Additionally, CDK5 was previously found in the cell’s nucleus [29,30]. Thus, it was proposed that CDK5 could have an impact also on gene expression. Taken together, we subsequently wondered whether CDK5 could affect the expression of clock genes. To investigate that aspect, we synchronized both wild-type and *Cdk5* ko cells with 100 µM forskolin and collected samples every 4 h starting from 12 up to 40 h after FST. We extracted total RNA and performed RT-qPCR to detect mRNA accumulation for circadian clock components. Our results showed that the lack of CDK5 affected clock genes in different ways. The first group of genes, such as *Arntl*, *Per1*, and *Cry1* (Figure 5A) or *Per2*, *Per3*, *Nr1d1*, and *Nr1d2* (Figure S3A), showed a substantial shift in their oscillation in the *Cdk5* ko cells, with a delay of about 2 to 4 h. The second group of genes formed by *Clock* and *Npas2* (Figure 5B) showed a higher amplitude in their oscillation in *Cdk5* ko compared to wild-type cells (de novo oscillation). Finally, *Cry2* showed a reduced amplitude in oscillation but higher expression (Figure 5C). All genes measured by RT-qPCR were also analyzed using CircaCompare [31] to evaluate, using a more parametric approach, the differential rhythmicity in terms of differences in the mesor, amplitude, and phase between wild-type and *Cdk5* ko cells (Table 1). Figure S3B shows the analysis performed on *Arntl*, *Per1*, and *Cry1* as an example. For *Arntl*, *Per1*, and *Cry1*, the phase differences were about 2 h, 3.7 h, and 2.7 h, respectively, confirming the previous observation. Altogether, these results indicate that CDK5 has a profound impact at the transcriptional level on clock genes.

All clock genes coding for the proteins involved in the main transcriptional–translational loop except for *Cry2* showed a delay in their mRNA expression. Additionally, our previous results showed that CDK5 affects the nuclear localization of CLOCK and ARNTL (BMAL1). In particular, ARNTL nuclear distribution in *Cdk5* ko cells was delayed by 4 h, mirroring the profile of mRNA induction of the clock genes. ARNTL is the main transcription factor promoting the expression of genes whose promoters contain E-boxes [32]. Thus, these observations raised the question of whether the different nuclear localization of ARNTL would mirror the various temporal recruitments of the transcriptional factor on the E-box of the clock gene promoters. Hence, we immunoprecipitated the transcription factor and performed RT-qPCR to amplify the E-box within the promoter of the *Per1* gene. Our results showed that the recruitment of ARNTL on the *Per1*_E-box was rhythmic in both cell lines. However, while the peak of rhythmicity in wild-type cells was 24 h after FST, it was 28 h after FST in the *Cdk5* ko cells (Figure S3C). Furthermore, we obtained the same result when we analyzed the recruitment of CLOCK on the same promoter region (Figure S3D). CircaCompare analysis confirmed the difference in phase between wild-type and *Cdk5* ko cells in the ARNTL (phase delay: circa 3.5 h) and CLOCK (phase delay: circa 4h) recruitment onto the E-box promoter region of *Per1* (Figure 5D,E, Table 2). Thus, our results showed that CDK5 influences the circadian period in the periphery via controlling nuclear accumulation and, therefore, temporal promoter occupancy of ARNTL on clock genes.

## 3. Discussion

Many pieces of evidence suggest that phosphorylation is essential for rhythmicity in circadian mammal models [13]. Furthermore, many kinases have been described to influence the circadian clock in living organisms in the past decades, such as Casein Kinase I, CDK1, and GSK3β [2]. The impact of Cyclin-dependent kinase 5 (CDK5) on the circadian clock was recently shown in the SCN, the pacemaker of circadian rhythmicity in mammals [20]. In mice with silenced *Cdk5* expression in the SCN, PER2 protein accumulation and nuclear translocation were severely affected. It appeared that CDK5 was the only kinase to stabilize PER2 and promote its nuclear import. Consequently, mutant mice had a shorter period than wild-type when their locomotor activity was measured in constant darkness. These observations resembled the phenotype of *Per2*^Brdm^ mice, where the *Per2* gene was bearing a deletion in the region where CDK5 phosphorylated it [33]. 

CDK5 was mainly described as part of the brain’s cytoskeleton or neuronal signaling molecules [34,35]. However, many observations indicated that CDK5 could play a pivotal role also outside the brain, such as in pancreatic β-cells [36], adipose tissue [37], ovary cells [38], and the male reproductive system [39,40]. Furthermore, circadian rhythms are observed and described at the peripheral level [41]. Therefore, we aimed to understand whether CDK5 could play a role in the peripheral circadian clock, using mouse embryonic fibroblasts as a molecular model. However, in our in vitro model, the picture differed from the one observed in the SCN. The lack of CDK5 led to a prolonged circadian period in mouse fibroblasts (Figure 1). These results are surprising, considering that the CDK5 kinase activity in embryonic fibroblast showed the same profile observed in the published results about the SCN, with a peak of activity during the subjective night (Figure 2E,F; [20]). Interestingly, our immunoprecipitation showed that CDK5 could temporally interact with PER1/2, CRY1, ARNTL (BMAL1), and CLOCK (Figure 3B). Our results suggest that this kinase is part of the central clock machinery. Other kinases are part of the complex, such as CKI [27], which directly phosphorylates PER1 and PER2 [42]. However, our results do not provide insight into the key element responsible for this complex formation. Many of these interactions might be indirect due to a specific clock protein working as a platform. To answer that question, the same immunoprecipitation should be performed using specific clock mutant cell lines to define the protein directly interacting with CDK5 to ensure the major complex formation.

Since CDK5 can virtually interact with many clock factors in the cell culture model, this observation raised the question of which factors could be responsible for the discordant results between the period observed in vivo [20] and in vitro (Figure 1). To investigate this aspect, we compared the impact of CDK5 on CLOCK and PER2. CDK5 can phosphorylate CLOCK at specific sites, Thr-451, Thr-461, and PER2 at SER-394, respectively [19,20]. However, CLOCK does not play a significant role in the central clock within the SCN, which is an essential difference with PER2 [43,44]. Our observations show that CDK5 can influence the nuclear localization of both CLOCK and PER2 in the cellular model in an opposite way. In wild-type cells, CLOCK nuclear accumulation shows a rhythmic profile while the rhythmicity was blunted in the *Cdk5* ko cells (Figure 4B, IB: CLOCK). More interestingly, CLOCK accumulated much more in the nuclei of the mutant cell line than the wild-type at each time point, mimicking an overexpression phenomenon. On the other hand, PER2 was regulated by CDK5, as observed in the SCN. The lack of this kinase strongly reduced PER2 accumulation, preventing the protein from entering the nuclei (Figure 4B, IB: PER2). 

As previously mentioned, similar to what was observed in the SCN, CDK5 activity was augmented in the subjective night phase (Figure 2), when the repressing complexes are present in the nucleus, including PER2 [45]. However, the CDK5 kinase activity profile is opposite to ARNTL and CLOCK transactivation, as measured by its chromatin binding activity (Figure 5D,E wt cells; Figure S3C,D wt cells). Since our results show that CDK5 regulates the CLOCK translocation into the nuclei, this enzyme’s kinase activity might also influence CLOCK recruitment onto the chromatin of E box-containing promoters. To confirm the hypothesis, we compared the CLOCK E box_*Per1* occupancy between wild-type and *Cdk5* ko cells, and we could observe a shift of about 4 h in the mutant cell line (Figure 5E and Figure S3D; ChIP: CLOCK). ARNTL recruitment onto the chromatin shifted about 3.5 h in the mutant cell line (Figure 5D and Figure S3C; ChIP: ARNTL). These observations agreed with what had previously been published since it is generally accepted that the heterodimer ARNTL: CLOCK is responsible for the transactivation in the cellular model [46,47]. Both proteins interact with CDK5 simultaneously (Figure 3), although the profile of their nuclear accumulation looks different (Figure 4B,C). The promoter occupancy of ARNTL in wild-type cells reflects its protein accumulation profile in the nuclei, and both shifted about 3.5 h in the *Cdk5* ko cells compared to the wild-type (Figure 4C, Figure 5D and Figure S3C). By contrast, CLOCK promoter occupancy does not mirror its total nuclear protein accumulation. In fact, CLOCK is accumulated in the nuclei for a more extended period than the one required for the transcriptional activity (Figure 4B, Figure 5E and Figure S3D). Thus, ARNTL is the limiting factor in the transactivating process, as previously suggested [48]. These results indicate that only a tiny pool of the total CLOCK protein might be directly involved in the transactivation.

In addition, the over-accumulation of CLOCK in the nuclei of *Cdk5* ko cells is not reflected in increased recruitment onto the chromatin (Figure 5E and Figure S3D, Table 2) or increased expression of clock genes (Figure 5A and Figure S3B, Table 1). Furthermore, the amount of BMAL1 recruited onto the E-box was slightly higher in the mutant cells (Figure 5D and Figure S3C, Table 2). Thus, our observations suggest that the *Cdk5* ko cells might somehow resemble the *Clock* Δ19 mutant mouse [46,49], which also has a long free-running period length [50]. The *Clock* Δ19 protein is missing part of its transcriptional activator domain and behaves as a dominant-negative repressor on multiple target genes. We speculate that in CDK5-deficient cells, too much CLOCK enters the nucleus, saturates all the heterodimers with ARNTL, and then the excess of the protein finally squelches additional transcriptional regulators from the repressive circadian complex. This scenario would explain the similar phenotype between *Cdk5* knock-out cells and *Clock* Δ19. If true, then the impact of CDK5 might rely on the nuclear import and stabilization of CLOCK as suggested [19], but the prolongation of the period length may be a consequence of overexpression, which changes unspecifically many processes in the cell by mass-action law.

Our evidence shows that CDK5 plays an essential role at the transcriptional level in regulating many genes driven by the circadian clock. CDK5 as a cotranscriptional regulator was already assumed before. For instance, it was observed that CDK5 could translocate into the nucleus upon neuronal stimulation and modulate the transcriptional repressor methyl-CpG-binding protein 2 [51]. Our results show that the lack of CDK5 delays the expression of the central clock genes for about 2 to 4 h (Figure 5A and Figure S3A,B, Table 1). Taken together, we demonstrated that a lack of CDK5 slows down the clock, delays ARNTL and CLOCK recruitment to E-boxes, and dysregulates circadian gene expression (Figure 5F). Our cellular model can virtually resemble the circadian clock observed in many peripheral organs. Therefore, it would be interesting to investigate how CDK5 can regulate the circadian clock in specific organs such as the liver, gut, muscles, and testis, which show independent oscillations [41].

With a more detailed analysis, it would be possible to link the circadian function of CDK5 with its other relevant functions in physiology outside the brain.

## 4. Materials and Methods

### 4.1. Cell Culture

Cells were cultured in Dulbecco’s modified Eagle’s medium (DMEM; PAN-Biotech, Aidenbach, Germany, No. P04-04510) containing 10% fetal calf serum (FCS) 100 U/mL penicillin-streptomycin. For real-time bioluminescence monitoring, NIH 3T3 cells in 3.5 cm dishes were co-transfected with an *Arntl* promoter-driven luciferase reporter gene and constructs expressing various shRNA against *CDK5*, as described [20]. The efficiency of these constructs had previously been tested [20]. Two days later, 100 nM dexamethasone was used to synchronize the circadian oscillators of the cells. After twice washing the dishes with DMEM, the cells were kept in phenol red-free DMEM with 5% fetal calf serum and 0.1 mM D-luciferin. Bioluminescence was recorded with a LumiCycle machine (Altimetrics, Wilmette, IL, USA) over three consecutive cycles. Using the LumiCycle analysis software (Altimetrics), the period length of each dish was determined using the inbuilt fast Fourier transform analysis. Experiments using the CDK5 inhibitor roscovitine (Merck KGaA, Darmstadt, Germany) were performed with 12 h pretreatment of the cells with the indicated amount of drug before measuring the bioluminescence, as described. NIH 3T3 cells without functional CDK5 were obtained using the CRISPR/Cas9 method. Briefly, proliferating NIH 3T3 cells were co-transfected with an expression vector for Cas9 and a guide RNA directed against *Cdk5* (mCDK5 guide RNA crRNA 1; GenScript Biotech Corporation, Piscataway, NJ, USA) cloned in frame with a linear puromycin resistance cassette vector. Clones of NIH 3T3 cells deficient for CDK5 were selected with 7 μg/mL puromycin and, subsequently, Western blot analysis for the lack of CDK5 expression. NIH 3T3 cells with and without CDK5 expression were induced for circadian rhythmicity with 10 µM forskolin [25]. Samples were collected at specific time points mentioned in the text.

### 4.2. Protein Extraction from Cells

Total protein was obtained from confluent cells plated in 10 cm dishes. The proteins were eluted with RIPA buffer (50 mM Tris-HCl pH7.4, 1% NP-40, 0.5% Na-deoxycholate, 0.1% SDS, 150 mM NaCl, 2 mM EDTA, 50 mM NaF) with freshly added protease and phosphatase inhibitors (Merck KGaA, Darmstadt, Germany), and homogenized using a disposable motor-driven pestle. Homogenates were kept on ice for 15 min and centrifuged for 15 min at 16,100× *g* at 4 °C. The supernatant was collected in new tubes. The protein concentration of the extracts was determined with the Bradford method (Biorad Laboratories Inc., Hercules, CA, USA) with BSA as standard.

### 4.3. Immunoprecipitation

Circa 400 µg of protein extract was diluted with the appropriate protein lysis buffer in a final volume of 250 µL and immunoprecipitated using the indicated antibody (ratio 1:50) at 4 °C overnight on a rotary shaker. The next day, samples were captured with 50 µL of 50% (*w*/*v*) protein-A agarose beads (Merck KGaA, Darmstadt, Germany) for 3 h at 4 °C on a rotary shaker. Before use, beads were washed three times with the appropriate protein buffer and resuspended in the same buffer (50% *w*/*v*). Next, the beads were collected by centrifugation and washed three times with NP-40 buffer (100 mM Tris-HCl pH 7.5, 150 mM NaCl, 2 mM EDTA, 0.1% NP-40). After the final wash, beads were resuspended in 2% SDS 10%, glycerol, 63 mM Trish-HCL pH 6.8, and proteins were eluted for 15 min at RT. Laemmli buffer was finally added, samples were boiled for 5 min at 95 °C and stored at −20 °C.

### 4.4. In Vitro Kinase Assay Using Immunoprecipitated Cdk5 from Synchronized Cells

The kinase activity of CDK5 in tissue culture cells was measured, as described [20]. Briefly, CDK5 was immunoprecipitated from 200 µg of protein extracts at different time points after forskolin synchronization. After immunoprecipitation at 4 °C overnight with 2× protein A agarose (Merck KGaA, Darmstadt, Germany), samples were diluted in washing buffer and split into two halves. One-half of the IP was used for the in vitro kinase assay. Briefly, 1 µg of histone H1 (Merck KGaA, Darmstadt, Germany) was added to the immunoprecipitated CDK5, and assays were carried out in reaction buffer (30 mM Hepes, pH 7.2, 10 mM MgCl_2_, and 1 mM DTT) containing [γ-^32^P] ATP (10 Ci) at room temperature for 1 h and then terminated by adding SDS sample buffer and boiling for 5 min. Samples were subjected to 15% SDS-PAGE, stained by Coomassie Brilliant Blue, and dried, and then phosphorylated histone H1 was detected by autoradiography. The other half of the IP was used for Western blotting to determine the total CDK5 immunoprecipitated from the SCN samples. The following formula was used to quantify the kinase activity at each time point: ([^32^P] H1/total H1 for each reaction)/CDK5 IP protein.

### 4.5. Nuclear/Cytoplasm Fractionation

Tissue culture cells were grown in 10 cm dishes. The cells were transferred to 1.5 mL Eppendorf tubes using 1 × PBS and centrifuged to obtain a cell pellet. First, the cytoplasm was obtained by incubating the cells for 10 min on ice in 90 μL of lysis buffer (10 mM EDTA, 1 mM EGTA, 10 mM Hepes pH 6.8, 0.2% Triton X-100, protease inhibitor cocktail; Merck KGaA, Darmstadt, Germany) and centrifuged at 2500× *g* for 5 min at 4 °C. The resulting supernatant was the cytoplasmic extract. Next, the pellet was washed three times with the lysis buffer and resuspended in 45 μL 1× NDB (20% glycerol, 20 mM Hepes pH 7.6, 0.2 mM EDTA, 2 mM DTT, protease inhibitor cocktail; Merck KGaA, Darmstadt, Germany) followed by adding 1 vol of 2× NUN (2 M Urea, 600 mM NaCl, 2% NP-40, 50 mM Hepes pH 7.6). After vortexing, the samples were incubated for 30 min on ice, centrifuged for 30 min at 13,000 rpm at 4 °C, and the supernatant was the nuclear extract.

### 4.6. Western Blot Analysis

The proteins were loaded onto a 10% SDS-PAGE gel and run at 100 V for 2 h. Once the migration was completed, the proteins were transferred to Hybond^®^ ECL™ nitrocellulose membranes for 1 h 30 min using a semidry blotting machine. The membrane was subsequently washed with 1× TBS 1×/0.1% Tween 20 (TBS-T) and blocked with 5% skim milk in TBST for 1 h. After washing, the membrane was stained with the appropriate primary antibody overnight. The antibodies against all the clock factors were described [52] (final dilution 1:1000). CDK5 was purchased from Cell Signaling Tech (1:1000), tubulin from Abcam (1:1000), and Lamin B from Santa Cruz (1:1000). The day after, membranes were washed three times with TBST followed by incubation with an HRP-conjugated secondary antibody for 1 h at room temperature and three more washing steps in TBST. HRP activity was detected with the WesternBright Sirius reagent (Advansta Inc., San Jose, CA, USA). The luminescence signal was digitally acquired with an Azure Biosystem Imager.

### 4.7. RNA Extraction and Real-Time-PCR

Cells grown in 6 cm Petri dishes were induced with 10 µM forskolin for the indicated time. Total RNA was extracted using the Nucleospin RNA II kit (Macherey-Nagel GmbH & Co. KG, Düren, Germany). In addition, 1 µg of total RNA was reverse-transcribed using Superscript II with random hexamer primers (Thermo Fisher Scientific, Waltham, MA, USA). Real-time PCR was performed using the KAPA probe fast universal master mix and the indicated primers on a Rotorgene 6000 machine (Qiagen, Hilden, Germany). The relative expression was calculated compared to the geometric mean of expression of the inert genes Sirt2, Nono, Tspo, and Tprkb, as described [53]. For a complete list of primers used in the paper, please see the list below (FAM: fluorescein, BHQ1: black hole quencher 1):

***Sirt2* (normalization probe)**
  FW: 5′-CAG GCC AGA CGG ACC CCT TC-3′
  RV: 5′-AGG CCA CGT CCC TGT AAG CC-3′
  TM: 5′-FAM-TGA TGG GCC TGG GAG GTG GCA TGG A-BHQ1-3′
***Nono* (normalization probe)**
  FW: 5′-TCT TTT CTC GGG ACG GTG GAG-3′
  RV: 5′-GTC TGC CTC GCA GTC CTC ACT-3′
  TM: 5′-FAM-CGT GCA GCG TCG CCC ATA CTC CGA GC-BHQ1-3′
***Tspo* (normalization probe)**
  FW: 5′-GGT CAG CTG GCT CTG AAC TG-3′
  RV: 5′-CAG TCG CCA CCC CAC TGA CA-3′
  TM: 5′-FAM-TGC CCG GCA GAT GGG CTG GGC-BHQ1-3′
***Tprkb* (normalization probe)**
  FW: 5′-GGC TGG CAT CAG ACC CAC AGA-3′
  RV: 5′-GGG CCC GTA GAG TCG GGA AA-3′
  TM: 5′-FAM-CCT GCG TCT GCC CTC TGA GGG CTG-BHQ1-3′
***Per1***
  FW: 5′-GCC CCG CCT CCT TGC TAC A-3′
  RV: 5′-ACT GGG GCC ACC TCC AGT TC-3′
  TM: 5′-FAM-TCC TTC CCT GCC AGT CCC CAA ACC CC-BHQ1-3′
***Per2***
  FW: 5′-TCC ACA GCT ACA CCA CCC CTT A-3′
  RV: 5′-TTT CTC CTC CAT GCA CTC CTG A-3′
  TM: 5′-FAM-CCG CTG CAC ACA CTC CAG GGC G-BHQ1-3′
***Per3***
  FW: 5′-CGT CTG GCA TCA GCC AGT GC-3′
  RV: 5′-CTC AGG GCC CAC GGC TTA CA-3′
  TM: 5′-FAM-CCT CTG GCC ACG CTC CGC CCC T-BHQ1-3′
***Cry1***
  FW: 5′-CTG GCG TGG AAG TCA TCG T-3′
  RV: 5′-CTG TCC GCC GAG TTC TAT G-3′
  TM: 5′-FAM-CGC ATT TCA CAT ACA CTG TAT GAC CTG GAC A-BHQ1-3′
***Cry2***
  FW: 5′-TGT CCC TTC CTG TGT GGA AGA-3′
  RV: 5′-GCT CCC AGC TTG GCT TGA-3′
  TM: 5′-FAM-CAG TCA CCC TGT GGC AGA GCC TGG-BHQ1-3′
***Arntl***
  FW: 5′-CCA AGA AAG TAT GGA CAC AGA CAA A-3′
  RV: 5′-GCA TTC TTG ATC CTT CCT TGG T-3′
  TM: 5′-FAM-TGA CCC TCA TGG AAG GTT AGA ATA TGC AGA A-BHQ1-3′
***Clock***
  FW: 5′-TTG CTC CAC GGG AAT CCT T-3′
  RV: 5′-GGA GGG AAA GTG CTC TGT TGT AG-3′
  TM: 5′-FAM-ACA CAG CTC ATC CTC TCT GCT GCC TTT C-BHQ1-3′
***Npas2***
  FW: 5′-CAG CCC TGA CTT CGG CCA TGA-3′
  RV: 5′-CAT CGC AGG ACC CAG GCA TCA-3′
  TM: 5′-FAM-CGG CAG CTC AGG CTG TTG CTG AGC C-BHQ1-3′
***Nr1d1***
  FW: 5′-GAA GTG TCT CTC CGT TGG CAT GTC T-3′
  RV: 5′-CGC TCT GCA TCT CGG CAA GCA T-3′
  TM: 5′-FAM-CTG TGC GTT TTG GGC GCA TCC CCA AG-BHQ1-3′
***Nr1d2***
  FW: 5′-GTG AGG GCC GCA CCC TGT-3′
  RV: 5′-CAG GGC TGG AGG CAG AGC T-3′
  TM: 5′-FAM-CCA GCG CCA TGG AGC TGA ACG CAG G-BHQ1-3′

### 4.8. Chromatin Immunoprecipitation

Chromatin immunoprecipitation from cells was performed, as previously described [26]. The cells were grown to confluency on 15 cm Petri dishes, induced with 10 µM forskolin, and fixed at the indicated time with 1% formaldehyde/1× phosphate-buffered saline buffer (PBS) for 10 min at 37 °C. At the end of the procedure, the input material and the immunoprecipitated DNA fragments were purified using a MinElute PCR fragment purification kit (Qiagen, Hilden, Germany). Real-time PCR reactions were performed using the KAPA probe fast universal master mix and the indicated primers on a Rotorgene 6000 machine. The efficiency of the precipitations was calculated by comparing the amount of precipitated material to the starting material.

*Per1*_E box
  FW: 5′-AGG CAC CAG AAA CCT CTT G-3′
  RV: 5′-GGC GTA GAT CTG ACA GGC TA-3′
  TM: 5′-FAM- TGC CAG AGT CTC CAA AGT ATG CCC AC-BHQ1-3′

### 4.9. Analysis of Circadian Rhythms (CircaCompare)

We performed the analysis described before [31]. The non-linear curve fit method was used to measure the differential rhythmicity of canonical clock genes between wild-type and *Cdk5* ko cells. Mean expression levels and standard deviations were calculated for each group separately. We estimated the loss or gain of rhythms and circadian properties, including amplitude, phase, and mesor, for each gene and genotype.

### 4.10. Statistical Analysis 

Statistical analysis of the experiments was performed using GraphPad Prism8 software. Values considered significantly different are highlighted by asterisks with * *p* < 0.05 and ** *p* < 0.01. 

## 5. Conclusions

Post-translational modifications are essential to fine-tune the mammalian circadian oscillator function. We found that the CDK5 had a circadian kinase activity profile in synchronized tissue culture cells. Additionally, we found CDK5 in a complex with multiple circadian clock components. In contrast to what was found in vivo, the lack of CDK5 provoked a lengthening of the free-running period. This discrepancy might be due to the improper accumulation of CLOCK protein in the nuclei. Understanding the exact contribution of CDK5 on the regulation of the circadian oscillator requires further investigation.

## Figures and Tables

**Table 1:** The expression of clock genes was analyzed using Circacompare [31]. The following parameters were analyzed: mesor, amplitude, phase, and the related *p*-values. AU: arbitrary units. 0.000 indicates a *p*-value < 0.001.

mRNA Accumulation	Delta Mesor (AU)	*p*-Value	Delta Amplitude (AU)	*p*-Value	Delta Phase (h)	*p*-Value
Arntl	−0.004	0.860	0.043	0.199	−2.02	0.000
Clock	NA	NA	NA	NA	NA	NA
Cry1	0.005	0.075	0.003	0.465	−3.71	0.000
Cry2	−0.108	0.000	0.005	0.765	−3.53	0.000
Npas2	0.001	0.107	−0.002	0.006	−1.41	0.194
Nr1d1	0.167	0.156	0.016	0.918	−2.55	0.000
Nr1d2	0.000	0.763	−0.001	0.687	−3.71	0.000
Per1	−0.031	0.011	0.006	0.680	−2.63	0.000
Per2	0.064	0.382	0.147	0.155	−2.76	0.000
Per3	0.036	0.058	0.030	0.238	−2.55	0.000

**Table 2:** ARNTL and CLOCK’s promoter occupancy on the Per1_Ebox was analyzed using Circacompare [31]. The following parameters were analyzed: mesor, amplitude, phase, and the related *p*-values. AU: arbitrary units. 0.000 indicates a *p*-value < 0.001.

ChIP	Delta Mesor (AU)	*p*-Value	Delta Amplitude (AU)	*p*-Value	Delta Phase (h)	*p*-Value
aARNTL	0.002	0.944	−0.021	0.491	−3.50	0.000
aCLOCK	−0.043	0.018	−0.012	0.622	−4.04	0.000

## Data Availability

Not applicable.

## Supplementary Materials

The Supplementary Materials are available online at https://www.mdpi.com/article/10.3390/clockssleep4010017/s1.

## References

[1] Schibler U., Sassone-Corsi P. (2002). A Web of Circadian Pacemakers. Cell.

[2] Brenna A., Albrecht U. (2020). Phosphorylation and Circadian Molecular Timing. Front. Physiol..

[3] Reppert S.M., Weaver D.R. (2002). Coordination of circadian timing in mammals. Nature.

[4] Dibner C., Schibler U., Albrecht U. (2010). The Mammalian Circadian Timing System: Organization and Coordination of Central and Peripheral Clocks. Annu. Rev. Physiol..

[5] Partch C.L., Green C.B., Takahashi J.S. (2014). Molecular architecture of the mammalian circadian clock. Trends Cell Biol..

[6] Kucera N., Schmalen I., Hennig S., Ollinger R., Strauss H.M., Grudziecki A., Wieczorek C., Kramer A., Wolf E. (2012). Unwinding the differences of the mammalian PERIOD clock proteins from crystal structure to cellular function. Proc. Natl. Acad. Sci. USA.

[7] Chiou Y.-Y., Yang Y., Rashid N., Ye R., Selby C.P., Sancar A. (2016). Mammalian Period represses and de-represses transcription by displacing CLOCK–BMAL1 from promoters in a Cryptochrome-dependent manner. Proc. Natl. Acad. Sci. USA.

[8] Buhr E.D., Takahashi J.S. (2013). Molecular Components of the Mammalian Circadian Clock. Handb. Exp. Pharmacol..

[9] Panda S., Antoch M.P., Miller B.H., Su A.I., Schook A.B., Straume M., Schultz P.G., Kay S.A., Takahashi J.S., Hogenesch J.B. (2002). Coordinated Transcription of Key Pathways in the Mouse by the Circadian Clock. Cell.

[10] Storch K.-F., Lipan O., Leykin I., Viswanathan N., Davis F.C., Wong W.H., Weitz C.J. (2002). Extensive and divergent circadian gene expression in liver and heart. Nature.

[11] Bellet M.M., Sassone-Corsi P. (2010). Mammalian circadian clock and metabolism–the epigenetic link. J. Cell Sci..

[12] Hirano A., Fu Y.H., Ptacek L.J. (2016). The intricate dance of post-translational modifications in the rhythm of life. Nat. Struct. Mol. Biol..

[13] Robles M.S., Humphrey S.J., Mann M. (2017). Phosphorylation Is a Central Mechanism for Circadian Control of Metabolism and Physiology. Cell Metab..

[14] Iwasaki H., Nishiwaki T., Kitayama Y., Nakajima M., Kondo T. (2002). KaiA-stimulated KaiC phosphorylation in circadian timing loops in cyanobacteria. Proc. Natl. Acad. Sci. USA.

[15] Tang D., Yeung J., Lee K.-Y., Matsushita M., Matsui H., Tomizawa K., Hatase O., Wang J.H. (1995). An Isoform of the Neuronal Cyclin-dependent Kinase 5 (Cdk5) Activator. J. Biol. Chem..

[16] Tsai L.-H., Delalle I., Caviness V.S., Chae T., Harlow E. (1994). p35 is a neural-specific regulatory subunit of cyclin-dependent kinase 5. Nature.

[17] Brinkkoetter P.T., Olivier P., Wu J.S., Henderson S., Krofft R.D., Pippin J.W., Hockenbery D., Roberts J.M., Shankland S.J. (2009). Cyclin I activates Cdk5 and regulates expression of Bcl-2 and Bcl-XL in postmitotic mouse cells. J. Clin. Investig..

[18] Kawauchi T. (2014). Cdk5 regulates multiple cellular events in neural development, function and disease. Dev. Growth Differ..

[19] Kwak Y., Jeong J., Lee S., Park Y.-U., Lee S.-A., Han D.-H., Kim J.-H., Ohshima T., Mikoshiba K., Suh Y.-H. (2013). Cyclin-dependent Kinase 5 (Cdk5) Regulates the Function of CLOCK Protein by Direct Phosphorylation. J. Biol. Chem..

[20] Brenna A., Olejniczak I., Chavan R., Ripperger J.A., Langmesser S., Cameroni E., Hu Z., De Virgilio C., Dengjel J., Albrecht U. (2019). Cyclin-dependent kinase 5 (CDK5) regulates the circadian clock. Elife.

[21] Contreras-Vallejos E., Utreras E., Gonzalez-Billault C. (2012). Going out of the brain: Non-nervous system physiological and pathological functions of Cdk5. Cell Signal..

[22] Pozo K., Bibb J.A. (2016). The Emerging Role of Cdk5 in Cancer. Trends Cancer.

[23] Sulli G., Lam M.T.Y., Panda S. (2019). Interplay between Circadian Clock and Cancer: New Frontiers for Cancer Treatment. Trends Cancer.

[24] Hsu F.-N., Chen M.-C., Lin K.-C., Peng Y.-T., Li P.-C., Lin E., Chiang M.-C., Hsieh J.-T., Lin H. (2013). Cyclin-dependent kinase 5 modulates STAT3 and androgen receptor activation through phosphorylation of Ser727 on STAT3 in prostate cancer cells. Am. J. Physiol. Metab..

[25] Yagita K., Okamura H. (1999). Forskolin induces circadian gene expression of rPer1, rPer2 and dbp in mammalian rat-1 fibroblasts. FEBS Lett..

[26] Tomov N., Surchev L., Wiedenmann C., Döbrössy M., Nikkhah G. (2019). Roscovitine, an experimental CDK5 inhibitor, causes delayed suppression of microglial, but not astroglial recruitment around intracerebral dopaminergic grafts. Exp. Neurol..

[27] Aryal R.P., Kwak P.B., Tamayo A.G., Gebert M., Chiu P.-L., Walz T., Weitz C.J. (2017). Macromolecular Assemblies of the Mammalian Circadian Clock. Mol. Cell.

[28] Wang Z., Wu Y., Li L., Su X.-D. (2012). Intermolecular recognition revealed by the complex structure of human CLOCK-BMAL1 basic helix-loop-helix domains with E-box DNA. Cell Res..

[29] Ino H., Chiba T. (1996). Intracellular localization of cyclin-dependent kinase 5 (CDK5) in mouse neuron: CDK5 is located in both nucleus and cytoplasm. Brain Res..

[30] Qu D., Li Q., Lim H.Y., Cheung N.S., Li R., Wang J.H., Qi R.Z. (2002). The protein SET binds the neuronal Cdk5 activator p35^nck5a^ and modulates Cdk5/p35^nck5a^ activity. J. Biol. Chem..

[31] Parsons R., Parsons R., Garner N., Oster H., Rawashdeh O. (2019). CircaCompare: A method to estimate and statistically support differences in mesor, amplitude, and phase, between circadian rhythms. Bioinformatics.

[32] Ripperger J.A., Schibler U. (2006). Rhythmic CLOCK-BMAL1 binding to multiple E-box motifs drives circadian Dbp transcription and chromatin transitions. Nat. Genet..

[33] Zheng B., Larkin D.W., Albrecht U., Sun Z.S., Sage M., Eichele G., Lee C.C., Bradley A. (1999). The mPer2 gene encodes a functional component of the mammalian circadian clock. Nature.

[34] Dhavan R., Tsai L.H. (2001). A decade of CDK5. Nat. Rev. Mol. Cell Biol..

[35] Dhariwala F.A., Rajadhyaksha M.S. (2008). An Unusual Member of the Cdk Family: Cdk5. Cell. Mol. Neurobiol..

[36] Wei F.-Y., Nagashima K., Ohshima T., Saheki Y., Lu Y.-F., Matsushita M., Yamada Y., Mikoshiba K., Seino Y., Matsui H. (2005). Cdk5-dependent regulation of glucose-stimulated insulin secretion. Nat. Med..

[37] Banks A.S., McAllister F.E., Camporez J.P., Zushin P.J., Jurczak M.J., Laznik-Bogoslavski D., Shulman G.I., Gygi S.P., Spiegelman B.M. (2015). An ERK/Cdk5 axis controls the diabetogenic actions of PPARgamma. Nature.

[38] Lee K.Y., Rosales J.L., Lee B.C., Chung S.H., Fukui Y., Lee N.S., Lee K.Y., Jeong Y.G. (2004). Cdk5/p35 expression in the mouse ovary. Mol. Cells.

[39] Musa F.R., Tokuda M., Kuwata Y., Ogawa T., Tomizawa K., Konishi R., Takenaka I., Hatase O. (1999). Expression of cyclin-dependent kinase 5 and associated cyclins in Leydig and Sertoli cells of the testis. J. Androl..

[40] Musa F.R., Takenaka I., Konishi R., Tokuda M. (2000). Effects of luteinizing hormone, follicle-stimulating hormone, and epidermal growth factor on expression and kinase activity of cyclin-dependent kinase 5 in Leydig TM3 and Sertoli TM4 cell lines. J. Androl..

[41] Richards J., Gumz M.L. (2012). Advances in understanding the peripheral circadian clocks. FASEB J..

[42] Camacho F., Cilio M., Guo Y., Virshup D.M., Patel K., Khorkova O., Styren S., Morse B., Yao Z., Keesler G.A. (2001). Human casein kinase Idelta phosphorylation of human circadian clock proteins period 1 and 2. FEBS Lett..

[43] DeBruyne J.P., Noton E., Lambert C.M., Maywood E.S., Weaver D.R., Reppert S.M. (2006). A Clock Shock: Mouse CLOCK Is Not Required for Circadian Oscillator Function. Neuron.

[44] DeBruyne J.P., Weaver D.R., Reppert S.M. (2007). CLOCK and NPAS2 have overlapping roles in the suprachiasmatic circadian clock. Nat. Neurosci..

[45] Koike N., Yoo S.-H., Huang H.-C., Kumar V., Lee C., Kim T.-K., Takahashi J.S. (2012). Transcriptional Architecture and Chromatin Landscape of the Core Circadian Clock in Mammals. Science.

[46] Gekakis N., Staknis D., Nguyen H.B., Davis F.C., Wilsbacher L.D., King D.P., Takahashi J.S., Weitz C.J. (1998). Role of the CLOCK protein in the mammalian circadian mechanism. Science.

[47] Shearman L.P., Sriram S., Weaver D.R., Maywood E.S., Chaves I., Zheng B., Kume K., Lee C.C., van der Horst G.T., Hastings M.H. (2000). Interacting Molecular Loops in the Mammalian Circadian Clock. Science.

[48] Kwon I., Lee J., Chang S.H., Jung N.C., Lee B.J., Son G.H., Kim K., Lee K.H. (2006). BMAL1 Shuttling Controls Transactivation and Degradation of the CLOCK/BMAL1 Heterodimer. Mol. Cell. Biol..

[49] Antoch M.P., Song E.-J., Chang A.-M., Vitaterna M.H., Zhao Y., Wilsbacher L.D., Sangoram A.M., King D.P., Pinto L.H., Takahashi J.S. (1997). Functional Identification of the Mouse Circadian Clock Gene by Transgenic BAC Rescue. Cell.

[50] Sujino M., Asakawa T., Nagano M., Koinuma S., Masumoto K.H., Shigeyoshi Y. (2018). CLOCKΔ19 mutation modifies the manner of synchrony among oscillation neurons in the suprachiasmatic nucleus. Sci. Rep..

[51] Liang Z., Ye T., Zhou X., Lai K.O., Fu A.K., Ip N.Y. (2015). Cdk5 Regulates Activity-Dependent Gene Expression and Dendrite Development. J. Neurosci..

[52] Schmutz I., Ripperger J.A., Baeriswyl-Aebischer S., Albrecht U. (2010). The mammalian clock component PERIOD2 coordinates circadian output by interaction with nuclear receptors. Genes Dev..

[53] Costa S.S.F., Wegmann D., Ripperger J.A. (2017). Normalisation against Circadian and Age-Related Disturbances Enables Robust Detection of Gene Expression Changes in Liver of Aged Mice. PLoS ONE.

