# Supplementary material for: Physical Interaction between Cyclin-Dependent Kinase 5 (CDK5) and Clock Factors Affects the Circadian Rhythmicity in Peripheral Oscillators
Source: Clocks Sleep. 2022 Mar 9;4(1):185–201. doi: 10.3390/clockssleep4010017 (PMC8946863; doi:10.3390/clockssleep4010017)
Supplement: Supplementary file 1 [file clockssleep-04-00017-s001.zip › clockssleep-1537662-supplementary.pdf]

Supplementary figures.

Physical interaction between Cyclin-dependent kinase 5 (CDK5) and clock factors affects the circadian rhythmicity in peripheral oscillators

Jürgen A. Ripperger[1], Rohit Chavan[1], Urs Albrecht[1], and Andrea Brenna[1,2*]

[1] Department of Biology, Faculty of Science and Medicine, University of Fribourg, 1700 Fribourg, Switzerland; juergenalexandereduard.ripperger@unifr.ch (JAR); rohitchavan27@gmail.com (RC); urs.albrecht@unifr.ch (UA)

[2] Department of Endocrinology, Metabolism, Cardiovascular System, Laboratory of Cardiovascular and Aging Research, Faculty of Science and Medicine, University of Fribourg, 1700 Fribourg, Switzerland

* Correspondence: andrea.brenna@unifr.ch

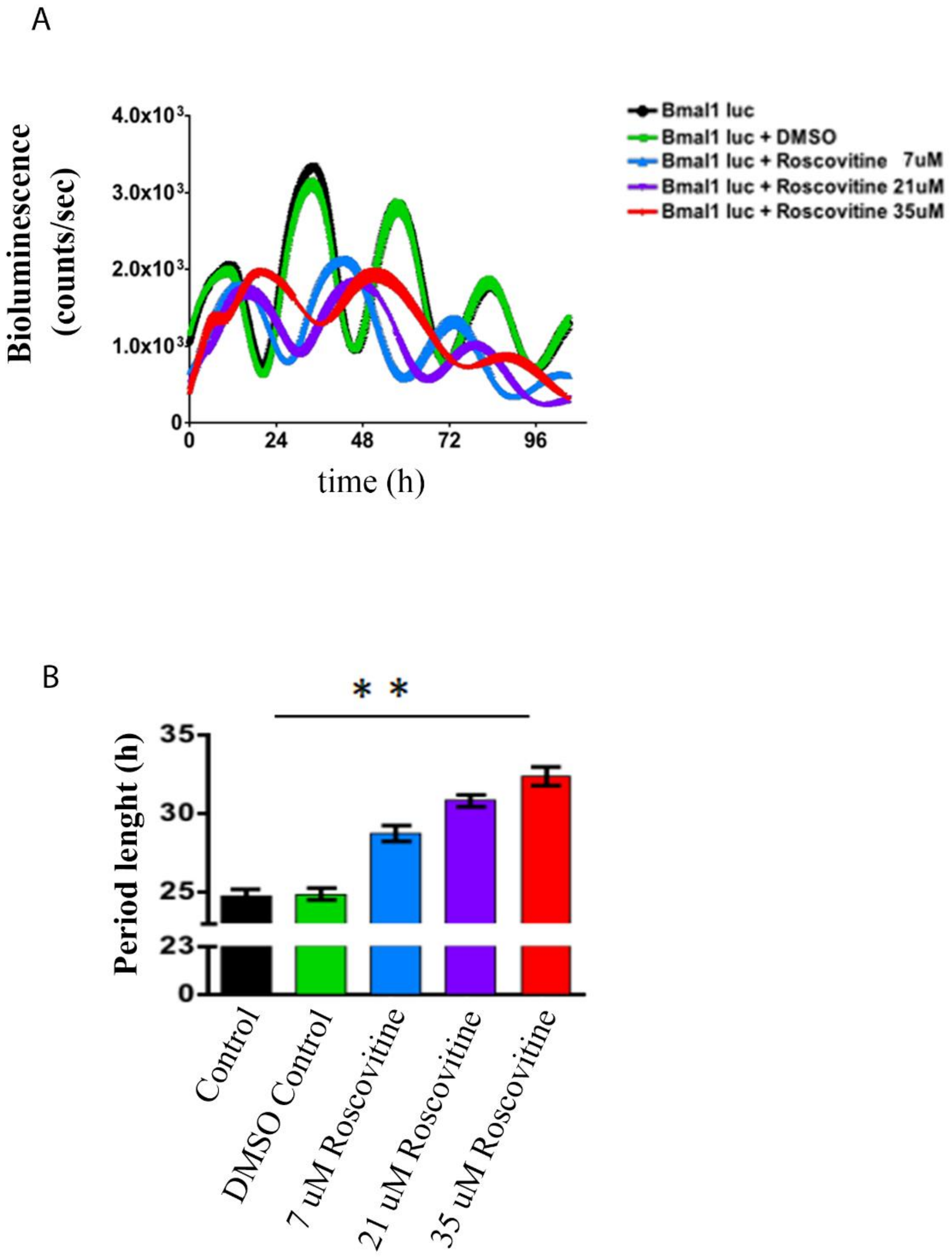
A
Bioluminescence (counts/sec)
4.0x10³
3.0x10³
2.0x10³
1.0x10³
0
0
24
48
72
96
time (h)
Bmal1 luc
Bmal1 luc + DMSO
Bmal1 luc + Roscovitine 7uM
Bmal1 luc + Roscovitine 21uM
Bmal1 luc + Roscovitine 35uM
B
* *
Period lenght (h)
35
30
25
23
0
Control
DMSO Control
7 uM Roscovitine
21 uM Roscovitine
35 uM Roscovitine

Figure S1

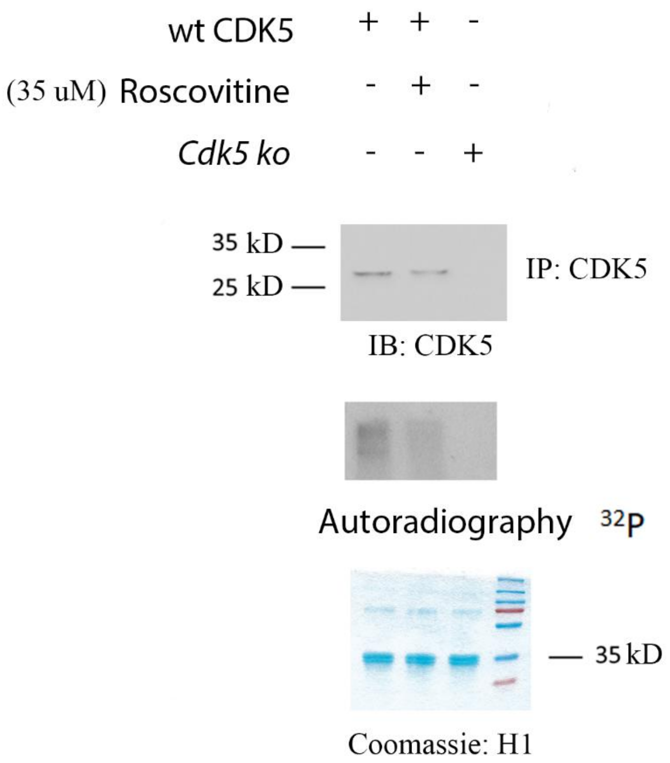

wt CDK5 + + -
(35 uM) Roscovitine - + -
Cdk5 ko - - +
35 kD
25 kD
IP: CDK5
IB: CDK5
Autoradiography 32P
35 kD
Coomassie: H1

Figure S2

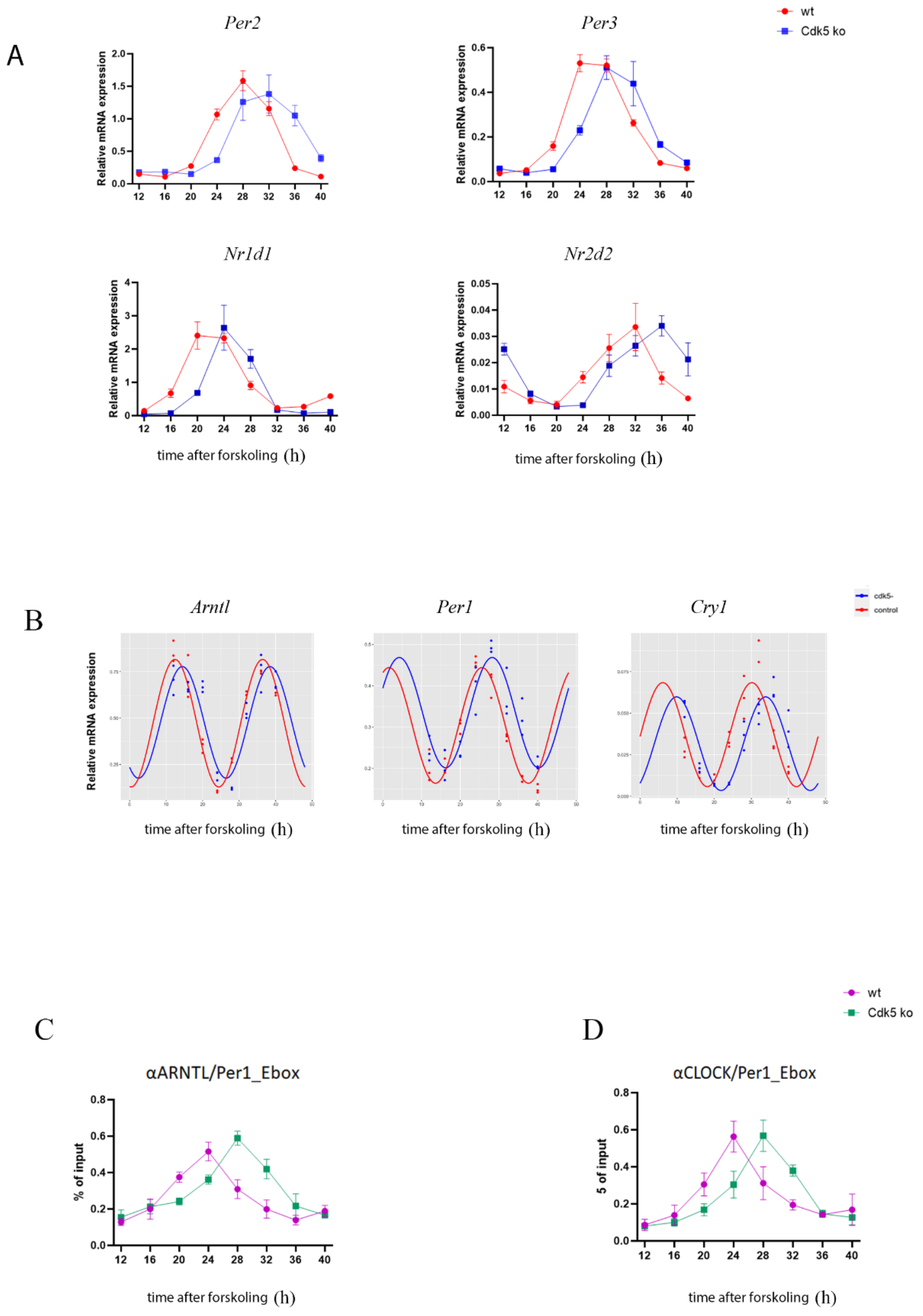

A
Per2
Per3
Nr1d1
Nr2d2
wt
Cdk5 ko
Relative mRNA expression
0.0
0.5
1.0
1.5
2.0
0.0
0.2
0.4
0.6
0
1
2
3
4
0.00
0.01
0.02
0.03
0.04
0.05
12
16
20
24
28
32
36
40
time after forskoling (h)
B
Arntl
Per1
Cry1
cdk5-
control
Relative mRNA expression
time after forskoling (h)
C
D
αARNTL/Per1_Ebox
αCLOCK/Per1_Ebox
wt
Cdk5 ko
% of input
5 of input
0.0
0.2
0.4
0.6
0.8
12
16
20
24
28
32
36
40
time after forskoling (h)

Figure S3

Figure S1. Roscovitine affects the circadian clock. A) NIH 3T3 cells transfected with an *Arntl*^{luc} reporter construct were treated for 12 h with an increasing amount of roscovitine (7, 21, 35 uM) and then synchronized with 100 nM dexamethasone. The bioluminescence was recorded over three consecutive days, n=3. B) The period length was quantified, and statistical analysis was performed. The results showed that an increase of roscovitine prolonged the period length of luciferase expression. Quantification of three independent experiments (n=3, mean ± SD). One-way ANOVA with Bonferroni's post-test. **: p<0.001

Figure S2. CDK5 kinase activity in the presence or absence of roscovitine. A) CDK5 was immune-precipitated from wild-type or *Cdk5* ko cells (negative control) samples, followed by an *in vitro* kinase assay using histone H1 as substrate in the presence of radiolabelled [γ ^{32}P]-ATP. In addition, the reactions using CDK5 from wild-type cells were performed in the absence or presence of 35 µM roscovitine. The experiment confirmed the specificity of the phosphorylated signal due to the CDK5 kinase activity.

Figure S3. CDK5 influences clock-controlled genes expression and ARNTL/CLOCK recruitment onto the *Per1* E-box containing promoter. A) A temporal profile of *Per2*, *Per3*, *N1rd1*, *N1rd2* mRNA induction was performed on samples obtained from wild-type and *Cdk5* ko cells. Quantification of three independent experiments (n=3, mean ± SD) was performed. B) Relative mRNA circadian rhythmicity was analyzed by CircaCompare [31]. For *Arntl* mRNA expression [Δ(*Cdk5* ko-wt]: Δmesor= -0.004 (AU, n.s.); Δamplitude= 0.043 (AU, n.s.); Δphase = -2.02 (h, p<0.001). For *Per1* mRNA expression [Δ(*Cdk5* ko-wt]: Δmesor= -0.031 (AU, n.s.); Δamplitude= 0.006 (AU, n.s.); Δphase = -2.62 (h, p<0.001). For *Cry1* mRNA [Δ(*Cdk5* ko-wt]: Δmesor= 0.005 (AU, n.s.); Δamplitude= 0.003 (AU, n.s.) ; Δphase = -3.71 (h, p<0.001). (C-D) Chromatin immunoprecipitation was performed using ARNTL (C) or CLOCK (D) antibody as bait. RT-qPCR was performed using specific probes that amplified the E-box region of *Per1*. Quantification of three independent experiments (n=3, mean ± SD) was performed.
